# Partial least squares based gene expression analysis in renal failure

**DOI:** 10.1186/1746-1596-9-137

**Published:** 2014-07-05

**Authors:** Shuang Ding, Yinhai Xu, Tingting Hao, Ping Ma

**Affiliations:** 1 Department of medical laboratory, The affiliated hospital of Xuzhou Medical College, No.99 Huaihaixi Road, Xuzhou 221000, China

**Keywords:** Renal failure, Partial least squares, Gene expression, Network

## Abstract

**Abstract:**

**Background**

Preventive and therapeutic options for renal failure are still limited. Gene expression profile analysis is powerful in the identification of biological differences between end stage renal failure patients and healthy controls. Previous studies mainly used variance/regression analysis without considering various biological, environmental factors. The purpose of this study is to investigate the gene expression difference between end stage renal failure patients and healthy controls with partial least squares (PLS) based analysis.

**Methods**

With gene expression data from the Gene Expression Omnibus database, we performed PLS analysis to identify differentially expressed genes. Enrichment and network analyses were also carried out to capture the molecular signatures of renal failure.

**Results**

We acquired 573 differentially expressed genes. Pathway and Gene Ontology items enrichment analysis revealed over-representation of dysregulated genes in various biological processes. Network analysis identified seven hub genes with degrees higher than 10, including *CAND1*, *CDK2*, *TP53*, *SMURF1*, *YWHAE*, *SRSF1*, and *RELA*. Proteins encoded by *CDK2*, *TP53*, and *RELA* have been associated with the progression of renal failure in previous studies.

**Conclusions**

Our findings shed light on expression character of renal failure patients with the hope to offer potential targets for future therapeutic studies.

**Virtual Slides:**

The virtual slide(s) for this article can be found here: http://www.diagnosticpathology.diagnomx.eu/vs/1450799302127207

## Background

Renal failure refers to the medical condition that kidneys fail to adequately filter waste products from blood. It is usually not reversible and patients with end stage renal failure have to be treated with long term dialysis or organ transplant [1,2]. Preventive and therapeutic options for this disease are still limited [3]. Capture the gene expression signature of end stage renal failure patients may enhance the development of novel therapeutic strategies.

High throughput microarray analysis is powerful to characterize the underlying pathogenesis of various diseases. Several studies have investigated the gene expression difference between renal failure patients and controls using this strategy [4-6]. These studies generally carried out variance or regression analysis to detect dysregulated genes. This statistical procedure ignored unaccounted array specific factors, including various biological, environmental factors. Previous studies [7,8] have suggested that partial least squares (PLS) based expression profile analysis is efficient in dealing with large amount of genes and fairly small samples. Compared with variance and regression analysis, PLS based analysis is more sensitive while maintaining reasonable high specificity, small false discovery rate and false non-discovery rate. Previous study using PLS analysis on other complex disease such as breast cancer has proved its feasibility [9]. Therefore, capturing the gene expression signature in renal failure patients by using PLS based analysis may provide new understanding of the pathogenesis and offer potential therapeutic targets.

In the current study, to investigate the gene expression difference between end stage renal failure patients and healthy controls, we performed PLS-based analysis by using gene expression data from the gene expression omnibus (GEO) database. Pathways or Gene Ontology items significantly over-represented with dysregulated genes were also acquired by using enrichment analysis. In addition, we constructed a protein-protein interaction (PPI) network with the proteins encoded by dysregulated genes to identify hub genes that may be related with disease progression.

## Methods

### Microarray data

The whole data set of gene expression profile GSE37171 from the GEO database was downloaded. This series represents transcription profile of 63 end-stage renal failure patients and 20 healthy controls. All samples were taken from peripheral blood. The dataset was based on the GPL570 platform ([HG-U133_Plus_2] Affymetrix Human Genome U133 Plus 2.0 Array). This study is approved by the institutional review board of the affiliated hospital of Xuzhou medical college (NO. 131081).

### Identification of differentially expressed genes

Normalization of raw intensity values was performed by using Robust Multi-array Analysis (RMA) [10]. The resulting log2-transformed expression value of each probe was used in subsequent analysis. A multivariate linear model was used to describe the relationship between gene expression values and the disease status. For each sample, the model is expressed as:

(1) $$y=\sum_{i=1}^{p}\alpha_{i}x_{i}+b$$

where *y* is the binary variable of disease status, 0 coded as control and 1 coded as renal failure; *p* is the total number of genes in the array. PLS analysis was then carried out to estimate the effects of each gene. The main purpose of PLS regression was to build orthogonal components (called ‘latent variables’ here). It is:

(2) $$\mathrm{COV}\left({t_{k}}^{,}u_{k}\to \max\right)$$

(3) $$\;\mathrm{Subject}\;\mathrm{to}||t_{k}||=1\;\mathrm{and}\;||u_{k}||=1$$

where ***t***_*k*_ is the *k*th latent variable decomposes from all individuals’ genes expression data *X* (the matrix of *n* × *p*, *n* refers to the number of individuals and *p* refers to the number of genes), ***u***_***k***_ is the *k*th latent variable decomposes from the phenotype data Y (*n ×* 1) [11]. The non-linear iterative partial least squares (NIPALS) algorithm [12] was used to calculate the PLS latent variables derived from the expression profile on the target trait, as follows:

1) Randomly initialize ***u***_0_ = ***Y***

2) ***w = X***^*T*^ ***u***_***0***_ ***, w = w/||w||***

3) ***t = Xw***

4) ***c = Y***^*T*^ ***t, c = c/||c||***

5) ***u = Yc***

6) if ***u***-***u***_**0**_ < 10E-8, go to step 7), else ***u***_**0**_ = ***u***, repeat step 2)-5)

7) ***X*** = ***X***-***tt***^T^ ***X***, ***Y*** = ***Y***-***tt***^T^ ***Y***

Then go back to 2) to calculate the next latent variable.

To evaluate the importance of the expressed genes on disease, the statistics of variable importance on the projection (VIP) [13] was calculated as:

(4) $$\mathrm{VI}P_{j}=\sqrt{\frac{p\sum_{k=1}^{h}\mathrm{Co}r^{2}\left(Y,t_{k}\right)w_{\mathrm{kj}}^{2}}{\sum_{k=1}^{h}\mathrm{Co}r^{2}\left(Y,t_{k}\right)}}$$

where, the Cor operator is the Pearson correlation coefficient, and for each ***w***_***k***_, it should be normalized by dividing ||*w*_*k*_||, and *h* is the number of latent variables used in the model.

To avoid the model over fitting, the best number of latent variables (*h* above) was determined by the prediction accuracy based on three folds cross validation. The *VIP* for each gene was then calculated with the *h* latent variables to obtain genes associated with renal failure. In addition, the false discovered rate (FDR) procedures were used to control the expected proportion of incorrectly rejected null hypotheses. The permutation procedure (*N =* 10000 times) was used to obtain the empirical distribution of PLS-based *VIP* in each replicate. The FDR for each gene was then calculated as:

(5) $$\mathrm{FD}R_{i}=\left(\sum_{j}^{10000}\sum_{i}^{p}\mathrm{Bool}\left(\mathrm{VI}P_{i,j}>\mathrm{VI}P_{i}\right)\right)/\left(10000p\right)$$

where Bool represents the logical value of expression: “True” codes as 1 and “False” codes as 0. Significant genes were selected with a threshold of FDR < 0.01.

### Enrichment analysis

Annotation of all probes was carried out by using the simple omnibus format in text (SOFT) files. To capture biologically relevant character of differentially expressed genes, enrichment analysis was implemented. All genes were firstly mapped to the Kyoto Encyclopedia of Genes and Genomes (KEGG) pathways (http://www.genome.jp/kegg/) [14] and Gene Ontology database [15]. Biological processes significantly overrepresented with differentially expressed genes were identified by using the hyper geometric distribution test.

### Network analysis

PPI is important for all biological processes since most protein function through its interaction with other proteins [16]. Among the proteins encoded by differentially expressed genes, those with more interactions with other proteins may play more important roles in the progression of renal failure. To visualize the interaction among these proteins and identify key molecules, a network was constructed by using the software Cytoscape (V 2.8.3, http://www.cytoscape.org/)[17]. The database (http://ftp.ncbi.nlm.nih.gov/gene/GeneRIF/) of NCBI was used to get the interaction information of all proteins. For each protein, the number of links (interactions) was defined as its degree. Proteins with degrees over 10 were selected as hub molecules in this study.

## Results

**Figure 1:**
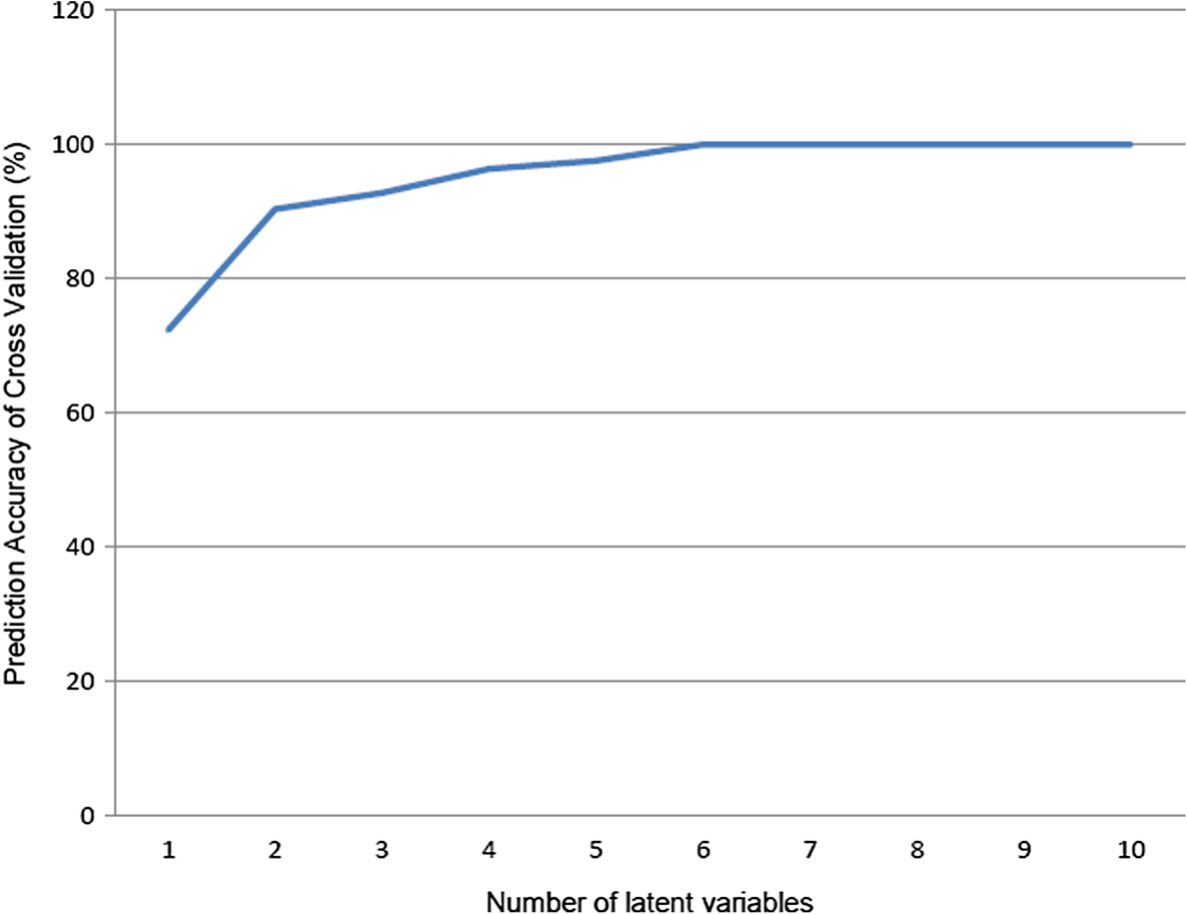
**The distribution prediction accuracy as the number of latent variable number increases.** The prediction accuracy achieves 100% when the latent variable number is six.

**Figure 2:**
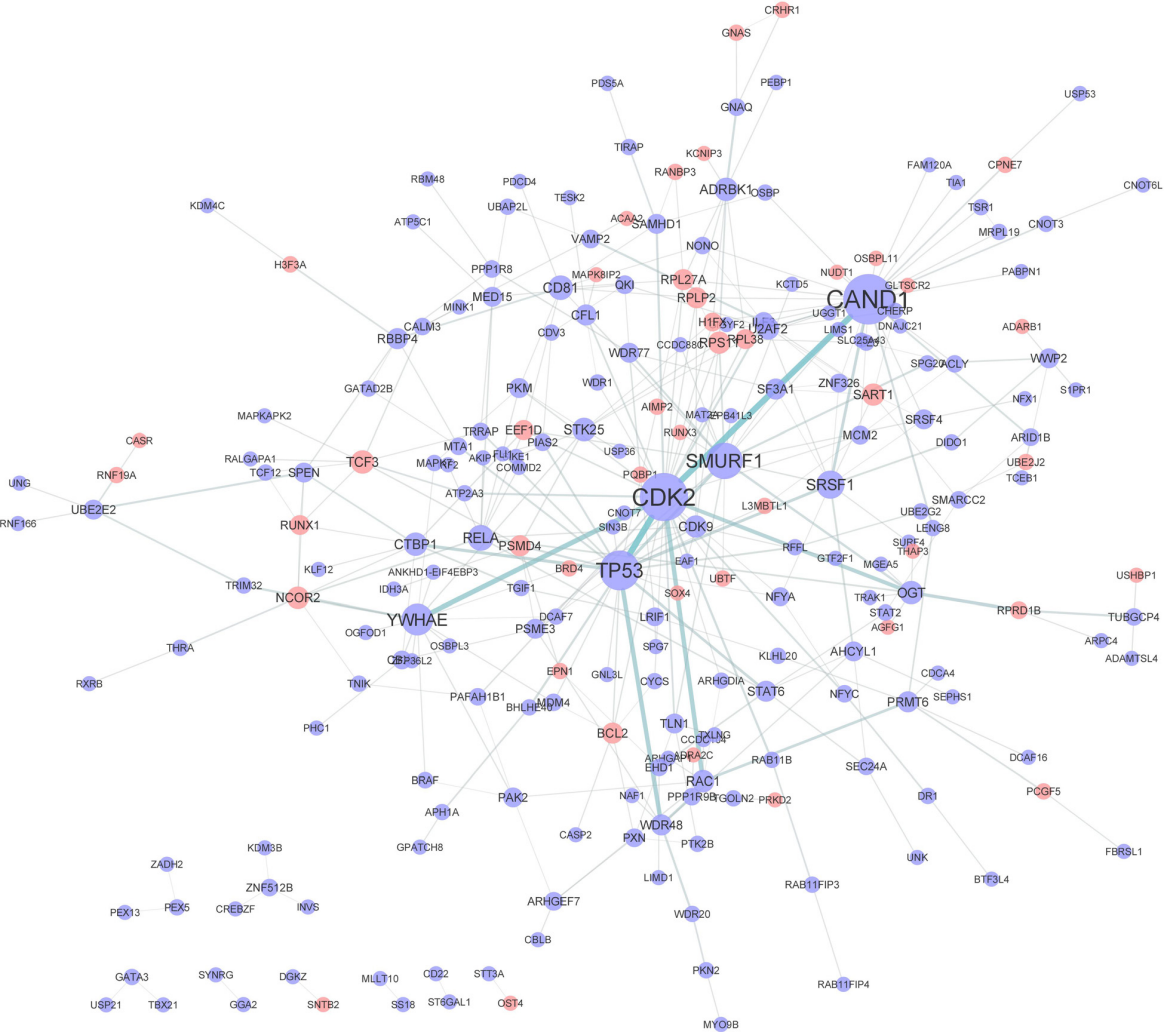
**Interaction network constructed by proteins encoded by differentially expressed genes.** Proteins with more interactions are shown in bigger size. Proteins in red are encoded by downregulated genes in patients while those in blue are encoded by upregulated genes in patients.

According to the prediction accuracy based on cross validation, six latent variables were used in the detection of differentially expressed genes (Figure 1). The results revealed that 573 genes were differentially expressed between end-stage renal failure patients and healthy controls, including 141 downregulated genes in patients and 432 upregulated ones. For all genes in the array, 6084 genes were mapped to various pathways, including 203 differentially expressed genes. The pathways enriched with differentially expressed genes are listed in Table 1. These pathways are involved in several systems, including nervous system, digestive system, and endocrine system. In addition, three cancer pathways, transcriptional misregulation in cancers (hsa05202), chronic myeloid leukemia (hsa05220) and small cell lung cancer (hsa05222) were also enriched with differentially expressed genes. A total of 16517 genes in the array were annotated based on the GO database, including 518 differentially expressed genes. Table 2 represents the five GO items enriched with selected genes. Protein binding (GO: 0005515) was the most significant GO item with over represented selected genes. In consistent with the pathway analysis, a transcription related GO item: transcription, DNA-dependent (GO: 0006351) was also identified to be overrepresented with dysregulated genes.Figure 2 illustrates the interaction network of proteins encoded by differentially expressed genes. Seven proteins, CAND1, CDK2, TP53, SMURF1, YWHAE, SRSF1, and RELA were identified to be hub molecules, with degrees of 31, 29, 22, 19, 15, 12, and 10 respectively.

**Table 1:** Pathways enriched with differentially expressed gene

**KEGG id**	**Pathway description**	**Pathway class**	**P-value**
hsa04722	Neurotrophin signaling pathway	Nervous system	2.09E-03
hsa05202	Transcriptional misregulation in cancers	Cancers	5.20E-03
hsa04120	Ubiquitin mediated proteolysis	Folding, sorting and degradation	5.22E-03
hsa05220	Chronic myeloid leukemia	Cancers	1.05E-02
hsa05222	Small cell lung cancer	Cancers	2.28E-02
hsa05010	Alzheimer's disease	Neurodegenerative diseases	2.43E-02
hsa04970	Salivary secretion	Digestive system	2.85E-02
hsa04130	SNARE interactions in vesicular transport	Folding, sorting and degradation	3.08E-02
hsa04912	GnRH signaling pathway	Endocrine system	3.17E-02
hsa04972	Pancreatic secretion	Digestive system	3.87E-02
hsa05100	Bacterial invasion of epithelial cells	Infectious diseases	4.05E-02
hsa04730	Long-term depression	Nervous system	4.60E-02

**Table 2:** GO items enriched with differentially expressed gene

**#GO id**	**GO description**	**GO class**	**FDR**
GO:0005515	protein binding	Function	2.92E-06
GO:0005730	nucleolus	Component	4.54E-05
GO:0005634	nucleus	Component	6.16E-05
GO:0006351	transcription, DNA-dependent	Process	4.19E-03
GO:0002326	B cell lineage commitment	Process	3.72E-02

## Discussion

Renal failure is a complex medical condition which may result from kidney injury or chronic diseases [18,19]. Microarray is a powerful technology for investigating the gene expression difference between end-stage renal failure patients and healthy controls. However, it is challenging to develop a suitable statistical model to deal with the small sample number and fairly large amount of genes. Previous studies on renal failure mainly used variance or regression analysis, without considering unaccounted array specific factors. Here we used PLS based analysis to identify dysregulated genes in end-stage renal failure patients.

Pathway enrichment analysis revealed that overrepresentation of dysregulated genes in various systems. Dysfunction of various systems may be complications of renal failure since kidneys are essential in the maintenance of homeostatic status. In addition, we also detected cancer-related pathways and GO items to be enriched with differentially expressed genes. The correlation between renal failure and cancer related biological processes may due to the dysfunction of cell cycle and DNA repair process in patients. Previous studies have demonstrated the enhanced expression of DNA repair-related proteins and induced cell cycle arrest at G1/S and G2/M in renal failure rats [20-22]. Overrepresentation of dysregulated genes in the chronic myeloid leukemia (hsa05220) pathway revealed the similar gene expression of these two diseases which may explain the causative effect of lymphocytic leukemia on renal failure [19]. These identified biological processes revealed the molecular signatures of renal failure.

To detect hub molecules, we constructed a network with proteins encoded by identified differentially expressed genes (Figure 2). Several hub molecules have been identified to play important roles in the progression of renal failure before. Take *RELA* for example, protein encoded by this gene is NF-kappaB p65. In consistent with our results, detection of NF-kappaB p65 based on immunohistochemical staining and ELISA suggested that NF-kappaB p65 in rat glomeruli of multiple organ failure was significantly higher than that of control group [23]. Attenuation of NF-kappaB p65 activation is effective in reducing endotoxic kidney injury [24]. Inhibition of inflammation through NF-κB also reduced renal dysfunction caused by sepsis in mice [25]. The involvement of NF-kappaB p65 in renal failure may be due to its interaction with inflammatory chemokines [26], such as CXCL16, which was increased in active nephrotic syndrome patients and correlated with blood lipids, urine protein and inflammation responses [27]. Genes involved in regulation of cell cycle, *TP53* and *CDK2*, were also identified as hub genes. Their involvements in renal failure through regulation of G1 cell cycle arrest were reported before [28]. Moreover, paricalcitol could prevent cisplatin-induced renal injury by suppressing the up regulation of *TP53* and *CDK2*[29]. Therefore, our study confirmed that these three genes may serve as potential targets for renal failure treatments. For the rest four hub genes, *SRSF1*, *CAND1*, *SMURF1*, and *YWHAE*, no previous report of their association with renal failure has been proposed before. Protein encoded by *SRSF1* is a member of the arginine/serine-rich splicing factor protein family. Up regulation of SRSF1 could increases the cellular pool of active p53 [30], suggesting the implication of SRSF1 in renal failure through its regulation of the p53. For *SMURF1*, protein encoded by this gene is an ubiquitin ligase that is specific for receptor-regulated SMAD proteins. It is reported that reduction of Smad7 due to the overexpression of Smurf1 in unilateral ureteral obstruction kidneys plays an important role in the progression of tubulointerstitial fibrosis [31], which a harmful process leading inevitably to renal function deterioration. Consistently, our analysis detected the up regulation of *SMURF1*, suggesting it may contribute to the progression of renal failure through its ubiquitination of SMAD7. Protein encoded by *YWHAE* belongs to the 14-3-3 family of proteins which mediate signal transduction by binding to phosphoserine-containing proteins. Quantitative protein expression profiling revealed that overexpression of YWHAE prompt the proliferation of renal cancer cells [32]. CAND1 may also promote the progression of renal cell carcinoma through its interaction with carbonic anhydrase IX [33]. Whether the up regulation contributes to the pathogenesis of renal failure needs further investigation.

## Conclusions

In summary, with gene expression profile downloaded from the GEO database, we carried out PLS based analysis to identify differentially expressed genes in end-stage renal failure patients and healthy controls. Pathway and GO enrichment analyses were also implemented to capture biological relevant characters. A network of proteins encoded by differentially expressed genes was constructed to identify key molecules. Our results facilitate the disclosure of the molecular mechanism underlying renal failure progression.

### Consent

Written informed consent was obtained from the patients for the publication of this report and any accompanying images.

## Competing interest

The authors declare that they have no competing interests.

## Authors’ contributions

PM designed the research and revised the manuscript. SD drafted the manuscript. SD, YX and TH carried out data analysis. All authors read and approved the final manuscript.
